# Genomic and Metabolomic Analysis of Antarctic Bacteria Revealed Culture and Elicitation Conditions for the Production of Antimicrobial Compounds

**DOI:** 10.3390/biom10050673

**Published:** 2020-04-27

**Authors:** Kattia Núñez-Montero, Damián Quezada-Solís, Zeinab G. Khalil, Robert J. Capon, Fernando D. Andreote, Leticia Barrientos

**Affiliations:** 1 Laboratory of Molecular Applied Biology, Center of Excellence in Translational Medicine, Universidad de La Frontera, Avenida Alemania 0458, Temuco 4810296, Chile; k.nunez03@ufromail.cl (K.N.-M.); d.quezada02@ufromail.cl (D.Q.-S.); 2 Scientific and Technological Bioresource Nucleus (BIOREN), Universidad de La Frontera, Temuco 4811230, Chile; 3 Biotechnology Investigation Center, Department of Biology, Instituto Tecnológico de Costa Rica, Cartago 159-7050, Costa Rica; 4 Institute for Molecular Bioscience, The University of Queensland, St Lucia, QLD 4072, Australia; z.khalil@imb.uq.edu.au (Z.G.K.); r.capon@imb.uq.edu.au (R.J.C.); 5 Department of Soil Science, “Luiz de Queiroz” College of Agriculture, University of São Paulo, Piracicaba, SP 13418-900, Brazil; fdandreo@gmail.com

**Keywords:** metabolomics, antibiotics discovery, secondary metabolites, cold-adapted bacteria, genome mining, silent gene clusters

## Abstract

Concern about finding new antibiotics against drug-resistant pathogens is increasing every year. Antarctic bacteria have been proposed as an unexplored source of bioactive metabolites; however, most biosynthetic gene clusters (BGCs) producing secondary metabolites remain silent under common culture conditions. Our work aimed to characterize elicitation conditions for the production of antibacterial secondary metabolites from 34 Antarctic bacterial strains based on MS/MS metabolomics and genome mining approaches. Bacterial strains were cultivated under different nutrient and elicitation conditions, including the addition of lipopolysaccharide (LPS), sodium nitroprusside (SNP), and coculture. Metabolomes were obtained by HPLC-QTOF-MS/MS and analyzed through molecular networking. Antibacterial activity was determined, and seven strains were selected for genome sequencing and analysis. Biosynthesis pathways were activated by all the elicitation treatments, which varies among strains and dependents of culture media. Increased antibacterial activity was observed for a few strains and addition of LPS was related with inhibition of Gram-negative pathogens. Antibiotic BGCs were found for all selected strains and the expressions of putative actinomycin, carotenoids, and bacillibactin were characterized by comparison of genomic and metabolomic data. This work established the use of promising new elicitors for bioprospection of Antarctic bacteria and highlights the importance of new “-omics” comparative approaches for drug discovery.

## 1. Introduction

The discovery of antimicrobial compounds is considered to be a major challenge for current and future public health due to the rising levels of multi drug-resistant infections [1]. Identification and characterization of bioactive compounds from natural resources led to the discovery of many commercially available drugs. Despite successes, the emergence of drug resistance (i.e., methicillin-resistant *Staphylococcus aureus*, vancomycin-resistant Enterococci) demands a relentless commitment to new drug leads [2]. Therefore, new strategies and next generation approaches are needed to increase the chances of drug discovery, particularly antibiotics [3,4,5]. 

Untapped environments have gained attention as a promising source of novel natural products [6,7,8]. Particularly extreme environments—such as the Antarctic—exhibit harsh conditions to survive, giving rise to complex ecosystems for the microorganism’s life. In this context, Antarctic bacteria are expected to harbor unique biosynthetic pathways, and as a result, new bioactive secondary metabolites resulting from evolution and adaptation to the polyextreme conditions that they are continuously facing [9,10]. 

In addition, genome mining has been used as an innovative tool to boost the discovery of antibiotics [11,12,13]. It has been shown that actinobacteria—the most productive group of bacteria in drug discovery—can possess dozens of biosynthetic gene clusters (BGCs) for the production of secondary metabolites, and it is estimated that only about 10% of them have been described [14]. Apart from this issue, culture-dependent techniques rely on the expression of those BGCs to detect, purify and characterize the secondary metabolites. Unfortunately, it is currently known that most of these pathways remain silent or cryptic under traditional culture conditions [15,16], being inaccessible for further studies. Since the expression of these genes is dependent on culture conditions, new methods to activate such clusters are needed to improve the exploration and exploitation of bacterial strains for the discovery of bioactive metabolites.

Previous studies reported by Capon et al. revealed that nitric oxide (NO) is intimately involved in the regulation of selected silent BGCs in both fungi and bacteria. More specifically, it was observed that endogenous/exogenous NO act as a transcriptional regulator of silent BGCs in the organism *Streptomyces* sp. CMB-M0423 [17]. Capon et al. discovered new methods for delivery of NO as a transcriptional regulator, including *in situ* generation of endogenous NO by natural chemical cues (i.e., a specific diketopiperazine (DKP) [18], and lipopolysaccharide (LPS) from Gram-negative bacteria) [19,20], as well as exogenous delivery of NO through addition of sodium nitroprusside (SNP) or 1% NO in N_2_ directly into microbial cultivations. These studies collectively revealed Nitric Oxide Mediated Transcriptional Activation (NOMETA) [18] microbial biodiscovery as a potentially cost effective and rapid approach for an *in situ* (i.e., non-genome mining) approach to accessing the valuable chemistry encoded within microbial silent BGCs [18]. In addition, Capon et al., developed and employed a high-throughput (HTP) 24-well plate microbioreactor method known as the MATRIX. The MATRIX allows rapid, reproducible one-milliliter cultivations of individual bacterial and fungal isolates under × 11 different media conditions, in broth shaken, broth static, and solid phase modes, to generate ×33 baseline extracts for chemical profiling per strain. These can then be repeated with various additives (i.e., NO) for a further ×33 profiles. It has been confirmed that MATRIX lead cultivations are scalable to preparative multi-flask/plate format [21,22].

Other multiple studies have recently focused on the activation/elicitation of silent BGCs by application of several techniques [23] including changes in temperature and nutrients [23,24,25], addition of antimicrobial and/or cytotoxic chemicals [26], coculture bacteria-bacteria and bacteria-fungi [27,28], genetic manipulation, and heterologous expression [29,30]. Though these methods have shown promising results for specific strains, all of them still have disadvantages for broad application, and the search for an ideal high-throughput method for activating silent BGCs continues [30]. Meanwhile, new -omics approaches and novel metadata analyses tools are recommended to provide new characterization of common natural sources. Among them, an accelerated drug discovery is reached when metabolomics and MS network indexing approaches are applied to prioritize strains before further scale-up [2,31]. Particularly, integrated culture-based strategies and genomic-driven approaches by comparative -omics might unfold new chemical classes of antibiotics [32]. With this in mind, our work aimed to characterize elicitation conditions for the production of antibacterial secondary metabolites—including silent BGCs—from Antarctic bacterial strains, based on MS/MS metabolomics and genome mining approaches. 

## 2. Materials and Methods 

### 2.1. Bacterial Isolation and Culture

Soil, marine water, and sediment samples were collected from different regions of the Antarctic peninsula on Antarctic Chilean expeditions during 2014 and 2016. Isolation of bacterial strains were conducted as follows. Samples were vigorously mixed and 1 g was exposed to 100 °C for 60 min for the selection of Actinobacteria, and other Gram positive and/or sporulated resistant bacteria, which are common producers of numerous secondary metabolites. After that, a dilution (10^−1^) was made under the following treatments during 30 min at 30 °C: phenol 1.5%, SDS 0.05%, Cloramine-T 1%, and saline solution (0.85% NaCl). Dilutions 10^−2^ and 10^−3^ for each sample treated were cultivated in soil extract medium (Antarctic soil 100 g/L and agar 18 g/L), oatmeal medium (oatmeal 60 g/L, agar 18 g/L) and R2A agar, all of them supplemented with nalidixic acid (25 μg/mL). Bacterial colonies were obtained and purified after incubation at 15 °C for a maximum of 30 days. A total of 34 Antarctic bacterial strains were selected for this study (Table S1). Inoculum for subsequent assays were obtained by culture of each strain on ISP-2 agar plates at 15 °C for 10 days. 

### 2.2. Screening of Culture Conditions for Elicitation of Secondary Metabolites

A colony of each bacterial strain was inoculated in 1.5 mL of twelve different media tested (Table S2) using a microbioreactor system (Applikon Biotechnology). An un-inoculated well for each medium was kept as a negative control. Cultures were incubated at 15 °C and 190 rpm for 7 days or until suitable growth. The extraction and detection of secondary metabolites were conducted as explained in the next section. Six culture media were selected (M2, IMA, ISP-4, CGA, YES, SE) as more suitable for secondary metabolites production. The cultures were repeated using selected media as previously described under three elicitation treatments: sodium nitroprusside (SNP 2 μM) as nitric oxide donor [18]; lipopolysaccharides from *Escherichia coli* O55:B5 (Merck LPS 0.6 nM); and coculture of two strains obtained from the same geographical region (Table S1). Addition of sterile water or spent-SNP (exposed to light for 10 days) were used as untreated controls for LPS and SNP, respectively. After 7 days of incubation at 15 °C and 190 rpm, extraction and detection of secondary metabolites were performed for each treatment. 

### 2.3. Extraction and Detection of Secondary Metabolites by LC-MS/MS 

Extraction of each culture was done *in situ* adding 2 mL of ethyl acetate (EtOAc). After incubation for 60 min at 190 rpm, organic phase containing secondary metabolites was collected, dried under N_2_ airflow, and resuspended in 20 μL of methanol to generate the analytes for LC-QTOF-MS/MS and to conduct antimicrobial assays. Aliquots of each extract (1 μL) were analyzed by LC-QTOF-MS/MS, using a Zorbax C_8_ RRHD 1.8 μm (2.1 × 50 mm) column, elution gradient of 2.50 min at 0.417 mL/min from 90% H_2_O/MeCN (acetonitrile) to 100% MeCN and 20 eV collision energy. 

### 2.4. LC-QTOF-MS/MS Metadata Analysis

Analysis and annotation of secondary metabolites (relevant adduct ions [M^+^H]^+^) were done by DEREPLICATOR statistical algorithm by the generation of molecular interactions and spectral networking using GNPS tool (*Global Natural Products Social Molecular Networking*) [31]. This analysis was performed for all the strains using the MS/MS metadata for each treatment with the following parameters: Precursor Ion Mass Tolerance = 0.02; Fragment Ion Mass Tolerance = 0.02; Minimal Pairs Cos = 0.5; Minimum Matched Peaks = 6; NodeTopk = 10; Minimum Cluster Size = 2; Run MSCluster = True; Library Min Match Peaks = 6, Library Search Score Threshold = 0.7; Search Analogs = true; Maximum Analog Search Mass Difference = 100Da; Filter St. Dev. Intensity = true, Min Fragment Ion Intensity: 0; Filter Precursor Ion Window = true, Filter Library = true; Filter Peaks in 50da Window = true. Visualization and data processing were done on Cytoscape 3.7.1. (The Cytoscape Consortium, California, CA, USA). Nodes belonging to control media were filtered in all data sets, and culture conditions for each node were determined. In the case of coculture, metabolites correspondent to each strain were determined by comparison of metabolites production when cultivated independently (pure culture); however, as this could not be accomplished for elicited metabolites, the whole set of compounds detected under coculture conditions was assumed a whole accessed diversity of compounds for this specific condition. All datasets included in this work are available at GNPS repository: ftp://massive.ucsd.edu/MSV000085261/.

### 2.5. Antibacterial Activity 

Crude extracts from each strain and treatment were screened for their inhibitory activity against known pathogens (*Escherichia coli* ATCC 22925, methicillin-resistant *Staphylococcus aureus* ATCC 25923, and *Enterococcus faecalis* CCCT 18.9) by disk diffusion assay as established by the Clinical & Laboratory Standards Institute (CLSI) [33,34]. Briefly, 15 µL of organic crude extract (60 µg/mL) was added to 6 mm‒Oxoid^TM^ Blank Antimicrobial Susceptibility Disks (Thermo Fisher Scientific Inc., Waltham, MA, USA) and dried at room temperature. Each disk was then placed onto Muller‒Hinton agar plates (BD Difco^TM^, Becton, Dickinson and Company, NY, USA) previously plated with a suspension of test pathogenic bacteria at 0.5 McFarland turbidity (equivalent to 1.5 × 10^8^ cells/mL). Diameters of inhibition zones were measured after 20 h of incubation at 37 °C. Ciprofloxacin, cefoxitin, and ampicillin were used as positive controls. 

### 2.6. Genome Sequencing and Assembly

Most productive strains based on secondary metabolites diversity and antibacterial activity were selected for genome sequencing (strains Dwa41.01, Se16.2.3, Se63.02, So1, So64.6, So13.3, SoD9). Selected bacteria colonies were used for genomic DNA extraction using UltraClean Microbial DNA Extraction Kit (MoBio Laboratories, Carlsbad, USA). DNA quality was assessed by fluorometry with QuantiFluor^®^ ONE dsDNA System (Promega) and determination of purity determining ratios 260/280 and 260/230. Library was prepared with the rapid sequencing kits SQK-RAD004 or SQK-RBK004 (Oxford Nanopore Technologies) and sequencing was performed in a minION platform (Oxford Nanopore Technologies) using the software MinKNOW. As well, DNA was sequenced on an Illumina HiSeq X ten sequencing platform using a paired-end library with average insert size of 350-bp followed by 2 × 150-bp sequencing. ONT-reads quality was verified with nanoPLOT [35], adapters and low-quality reads (quality > 10, length > 5000bp) were trimmed with Porechop y NanoFilt [35], respectively. Illumina-reads were trimmed with Fastp [36] using default parameters and verified with FASTQC [37]. *De novo* assembly was accomplished with the software Unicycler [38] and the default parameters for hybrid assembly. When necessary an additional finalization of the assembly was done using the tool FinisherSC [39] and *quickmerge* [40] to close the genome before and after Pilon polishing [41]. Quality for each assembly was assessed with Quast [42], and completeness/contamination were determined with CheckM [43] and miComplete [44]. This Whole Genome Shotgun project has been deposited at DDBJ/ENA/GenBank under the bioproject accessions PRJNA605861 PRJNA445286, including the genomes CP050451 (strain Se16.2.3), CP050452 (strain SoD9b), CP050453 (strain Se63.02b), CP050454 (strain Dwa41.01b), CP049924 (strain So1b), CP048817 (strain So64.6b), CP048835 (So13.3). 

### 2.7. Genome Analysis and Identification of Secondary Metabolites

Determination of coding sequences, RNAs and type of genes was done with the annotation tools NCBI Prokaryotic Genome Annotation Pipeline (2013) [45] and RAST [46]. The closest taxonomic group for each strain was identified using the 16S rRNA nucleotide sequence for comparison in BLASTN (http://www.ncbi.nlm.nih.gov/blast) using DDBJ/EMBL/GenBank nucleotide sequence databases. The phylogenetic tree was done after alignment of the most similar 16S rRNA sequences using Mafft version 7 [47] L-INS-i method, followed by a maximum likelihood (ML) phylogenetic tree construction. The Akaike information criterion [48] was applied to find the most accurate substitution model method using IQ-TREE web server [49]. Finally, phylogenetic tree was build with SeaView version 4 [50] using ML with the following parameters: GTR substitution model, 1000 bootstrapped data sets, 4 substitution rate categories for across site rate variation, estimated gamma distribution parameter, optimized variable sites and empirical nucleotide equilibrium frequencies, heuristic search of starter tree with BioNJ algorithms, and tree topology search with NNIs. Biosynthetic gene clusters (BGCs) corresponding with the production of secondary metabolites for each strain were detected and characterized using antiSMASH 5.1.0. [51] and the closest known BGC were assigned whether matches were found when compared with the Repository of Known Biosynthetic Gene Clusters MiBiG. This data was manually compared with metabolomics results from LC-QTOF-MS/MS analysis.

## 3. Results

### 3.1. Screening of Multiple Nutritional Conditions is Recommended to Access a Major Diversity of Secondary Metabolites

**Figure 1:**
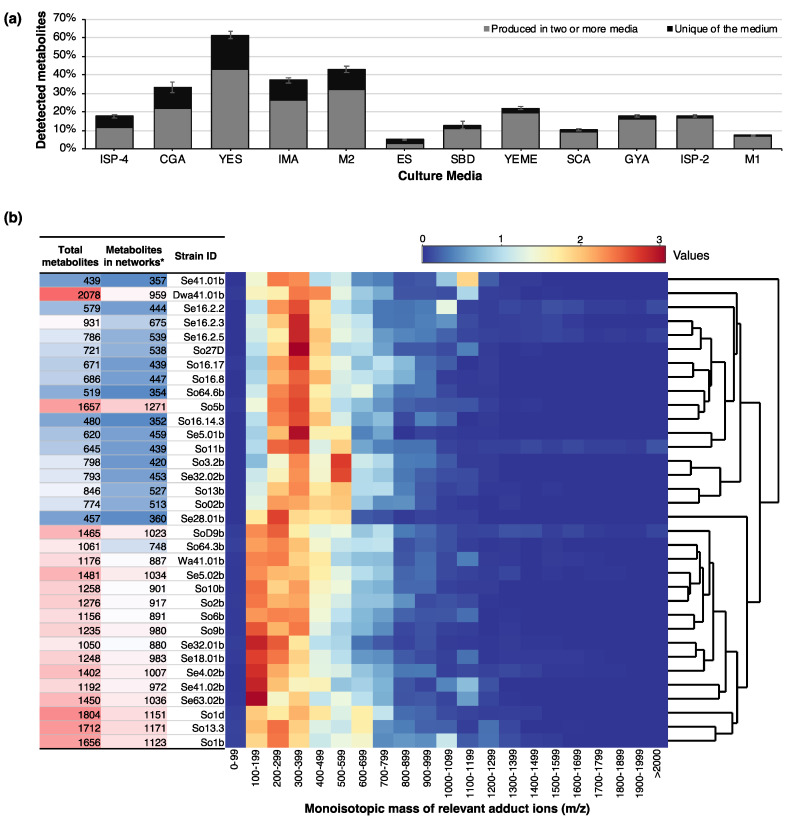
Summary of metabolomic results based on LC-QTOF-MS/MS detection and obtained from 34 Antarctic bacteria strains exposed to different culture conditions. (**a**) Mean percentage of adduct ions detected for Antarctic bacteria strains in different culture media; (**b**) Hierarchical clustering by UPGMA using Euclidean distance method for the monoisotopic mass (*m/z*) distribution of metabolites (adduct ions) detected for each bacterial strain in the sum of conditions tested. Heatmap represents the amount of counts per range normalized by strain. Table on the left shows the total number of adduct ions detected and those forming networks (*: ≥ 3 nodes) after networking analysis by GNPS (Global Natural Products Social Molecular Networking).

The MS/MS metadata analysis showed the total metabolites (relevant adduct ions) produced under the different conditions screened, allowing us to determine the culture media conditions that produce a greater diversity of metabolites (Figure 1a). Using this information, six essential culture media were selected, which produce at least 95% of the detected metabolites. Taking this into account, subsequent analyses were performed using the culture media M1, CGA, IMA, ISP-4, YES, and ES (Table S2), which allowed the obtention of the greatest diversity of metabolites. Moreover, this selection carries biological sense considering that these six culture media include the majority of nutritional combinations for the biosynthesis of secondary metabolites, such as diverse carbon sources (glucose, glycerol, mannitol, sucrose, maltose); addition of different salts and the presence/absence of other vitaminized compounds and sources of amino acids (malt extract and yeast extract). The results also revealed that by using a set of culture media, we were able to increase the number of metabolites produced under one traditional growth medium in more than 100% within the assessed bacteria. Despite the fact that this method has been documented as the simplest form of activating silent metabolites and has proven to be useful in the discovery of multiple bioactive secondary metabolites [52], most of the recent studies characterize the biological activity and compounds production only under a single culture condition.

**Figure 2:**
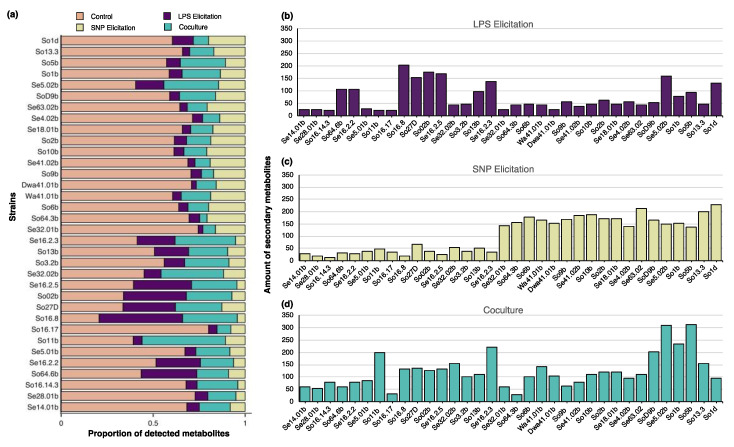
Metabolites (adduct ions) detected by HPLC-QTOF-MS/MS for 34 Antarctic bacteria cultivated under different elicitation treatments. (**a**) Proportion of metabolites detected for each elicitation treatment; (**b**) number of metabolites elicited by the addition of LPS (lipopolysaccharide) to the culture; (**c**) number of metabolites elicited by the addition of SNP (sodium nitroprusside) to the culture; (**d**) number of metabolites elicited by coculture of two Antarctic strains.

With the selected culture media, 34 strains were grown under control conditions (basal production of metabolites) and using elicitation methods such as the addition of LPS or SNP, or through coculture of two strains isolated from the same sample or geographic region. The metabolites produced by each Antarctic strain under the assessed culture conditions were detected through high-resolution tandem mass spectrometry (QTOF-MS/MS), obtaining the monoisotopic mass of the relevant adduct ions present on each sample, and its fragmentation pattern as well. The identification of the metabolites produced under each assessed condition was performed by massive data analysis using dereplication and molecular networking. The results showed that all conditions, including culture media and elicitation treatments, generate a broad range of unique metabolites in variable amounts depending on the evaluated strain (Figure 1b and Figure 2). The composition and concentration of nutrients have an effect over the global regulatory mechanisms in bacteria [53] and the variety of metabolites detected could have been originated by different base compounds or “*building blocks*”, which vary depending on the sources of nitrogen, carbon and phosphorous available for each bacteria; as it happens in the activation of particular metabolic pathways in response to the limitation of some specific nutrients on each media, which might be interpreted as the presence of competitors [54]. It was not possible to define any of the six culture media as the best metabolite producer for all the set of strains.

Within the evaluated bacteria, the number of detected metabolites ranged from 438 to 1808 in the sum of culture conditions assessed for each strain (Figure 1b). This outcome was as expected, since even though previous reports state that the genetic clusters associated with the production of secondary metabolites (BCGs) within the Actinobacteria range from 20 to 30 [55], each type of metabolic pathway may vary in its base compounds, enzymatic mechanisms, and modification reactions, responding to the environmental signals so that a wide variety of different compounds are achieved (i.e., metabolic intermediates) from a single biosynthetic pathway [56]. Most detected metabolites showed a monoisotopic mass that ranged from 100 to 500 *m/z* (Figure 1b) and in some cases, a large amount of compounds with a higher mass (around 1000 *m/z*) was observed (Figure 1b), which are of particular interest since they correspond to high complexity molecules, presenting more possibilities of chemical modifications that generate a major amount of analogues, thereby increasing the probabilities of identifying novel molecules.

On the other hand, the molecular networking analysis allowed for the spectral visualization of the whole set of relevant adduct ions detected by the LC-QTOF-MS/MS, and it was used to determine under which culture conditions are those metabolites produced for each strain. Related molecules are presented as maps of nodes related to each other through a similarity statistic cosine score (>0.5) [57]. For all the strains, metabolites networks were found (Figure 1b). The presence of metabolites conforming molecular networks represents molecules with some degree of similarity, through which it is possible to elucidate structures more efficiently and obtain reclusive information about the number of analogues and/or multimers that can be obtained from the assessed strain. In this study, two groups of strains were defined (Figure 1b) based on an UPGMA clustering analysis using the number of detected metabolites and their mass distribution, where 16 strains fit in a profile of a large number of metabolites detected, most of them forming networks. However, the second group are more likely to produce high-molecular weight metabolites based on their greater number of adduct ions with high monoisotopic mass.

### 3.2. Response to Elicitation Treatment Varies among Strains

In 100% of the evaluated strains, activation was observed in the production of metabolites for all the elicitation treatments (addition of LPS, addition of SNP and coculture). The metabolites detected only under elicitation treatments (forming networks) represented on average a 42%, ranging from 20% (strain So16.17) to 79% (strain So16.8) of the total metabolites produced. Results were variable for each strain, thereby demonstrating that some of the elicitation treatments resulted in higher activation of metabolites. In spite of this, each one of the different assessed strains shown a remarkably higher response in one of the treatments (Figure 2); only in few cases, a similar number of elicited metabolites was observed in the 3 of them (strains Se5.01b, So16.17, So16.14.3, Se28.01b and Se14.01b).

#### 3.2.1. Sodium Nitroprusside (SNP)

Regarding the activation using SNP as a nitric oxide donor [19], the result is of great importance in the context of the recent efforts made in the study of novel silent secondary metabolites from Actinobacteria [58], highlighting the ability of this chemical compound to elicit multiple metabolites in all the assessed strains, from which, at least 12 out of 34 strains displayed a significantly higher amount of activated metabolites compared to the elicitation treatments using LPS and coculture (Figure 2). Nitric oxide acts as a cell damage indicator, mediating inflammatory responses in mammal cells; it has also been shown to stimulate the production of secondary metabolites in fungi [19] and increase the accumulation of metabolites in vegetal cells [59]. The results herein point out the possibility that some Antarctic bacteria regulates a major part of their secondary metabolites, sensing environmental signals from nitric oxide. These bacteria might even represent a good model for studying this recently proposed response to SNP [18].

**Figure 3:**
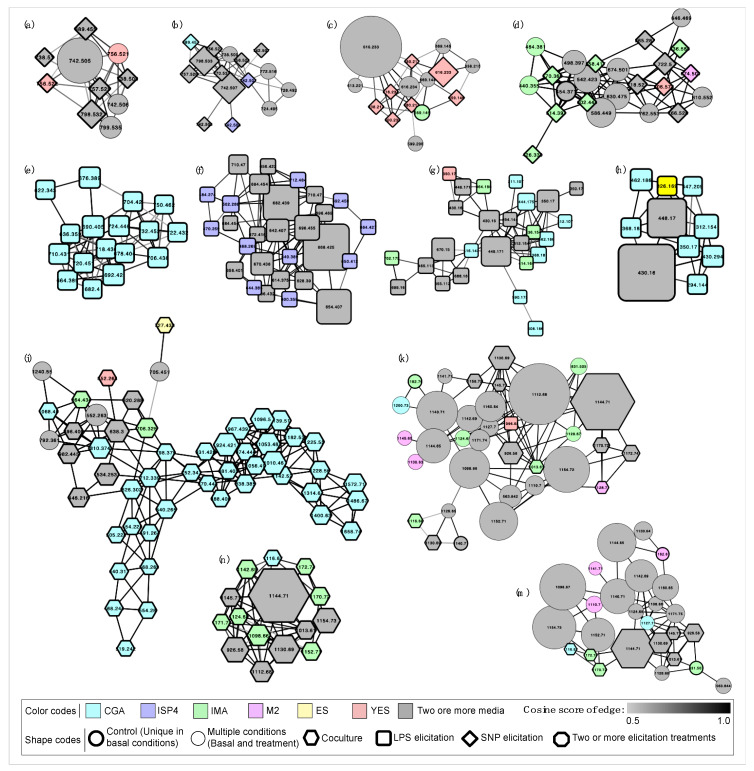
Molecular networking of adduct ions produced by Antarctic bacteria strains in different culture conditions and obtained by HPLC-QTOF-MS/MS metadata analysis through GNPS (Global Natural Products Social Molecular Networking). Networks belongs to the metabolome of the strains: (**a**) Dwa41.01b; (**b**) So1d; (**c**) So2b; (**d**) So10b; (**e**) Se5.02b; (**f**) Se16.2.3; (**g**) So16.8; (**h**) So13b; (**i**) So11b; (**k**) Se63.02b; (**n**) Dwa41.01b; (**m**) Se18.01b. Each spectrum is shown as a node labelled with the monoisotopic mass (*m/z* [M^+^H]^+^), whose size is proportional to the abundance. Related metabolites are linked by edges with cosine scores between 0.5 and 1. Elicitation treatments and culture conditions are represented as shown in the legend based on the color and shape of the node.

The network analysis showed that some strains produce molecular networks constituted mostly or completely by metabolites elicited by the addition of nitric oxide (SNP) (Figure 3a–d). Some of these metabolites seem to be elicited depending on the culture media used to grow the bacteria. Notably, there are some metabolites detected in large amounts, as is the case with the strains Dwa41.01b (Figure 3a), So1d (Figure 3b), and So2b (Figure 3c), since these might play an essential role in response to the applied treatment, thus being more abundant.

#### 3.2.2. Lipopolysaccharide (LPS)

The gene expression associated with the production of natural products in microorganisms depends directly or indirectly on environmental signals and/or physical interactions with organisms from the same habitat. There are multiple approaches to replicate these conditions and activate silent BGCs, exhibiting differences in their success rate depending on the organism being evaluated [60]. Particularly, the LPS is part of the outer membrane of Gram-negative bacteria, and since it is an exposed element (in non-encapsulated organisms), plays a fundamental role in recognition during bacterial colonization. Due to this, it has been used as an inductor agent of immune system responses in mammals [61,62] and plants [63], allowing in many instances the discovery of secondary metabolites with anti-inflammatory activity [64]. Also, the stimulation of secondary metabolites using LPS in fungi has been recently described, suggesting the possibility that the coculture between fungi-bacteria might not be necessary for the obtention of these molecules [19,65,66].

The effect of the LPS in bacterial cultures has not been described or compared to the traditional culture conditions in terms of metabolites production. In this work, the elicitation of metabolites using LPS was evidenced in 100% of the cultures. Particularly, when compared with other treatments (i.e., SNP and coculture), 7 out of 34 strains largely increased their production of secondary metabolites (Figure 2a). Most of the detected molecules activated through LPS stand out because they constitute networks of multiple metabolites in high abundance, and others are conforming too many groups of metabolites with high monoisotopic mass (Figure 3e–h). In particular, the high abundance of certain activated metabolites indicates overexpression of these compounds, and therefore, the key role that they may play in response to LPS present in the environment.

#### 3.2.3. Coculture

Detected metabolites by activation under the condition of coculture—presented in all the assessed strains—were found mostly conforming networks of numerous adduct ions (Figure 3i–m). Under this condition, it is not possible to understand or explain which factor is triggering the metabolic response, since it corresponds to the generation of a complex system of multiple interactions between two living organisms competing or collaborating with each other for their growth and media colonization. In their natural environment, the microorganism communities are exposed to intra- and interspecies interactions that might have beneficial or harmful consequences for the organisms involved in such interactions. The compounds’ diversity and the conditions that activate specific BGCs happening naturally are almost completely unknown [67]. As a result, the coculture has been the method most frequently used to achieve the identification of novel silent secondary metabolites, and have permitted the discovery of different compounds with antibiotic activity within bacteria of the genera *Streptomyces* [52,68].

It has been supposed that the existing wide diversity of metabolites was originated from the interspecies interactions between organisms that coexist in the same environment [52], experimental results have showed that bacteria isolated from the same location are much more efficient inhibiting each other [69,70]. Also, as has been demonstrated, direct physical contact with other organisms varies the expression of secondary metabolites in Actinobacteria [71]. Due to this, the result obtained in the coculture of Antarctic strains isolated from the same environment generated the expected positive outcome in the elicitation of secondary metabolites. The possibilities of obtaining elicitation through coculture could be greater than through other treatments. Nevertheless, a disadvantage is the unknown type of interactions and signals generated during this elicitation process, which implies that additional precautions must be taken into account to ensure the reproducibility of the assay since minimal variations in the inoculum density or in the culture conditions might alter the relations and chemical responses of the bacteria in coculture. In addition, beyond the drug discovery, further genome mining and gene expression studies are needed in cases where a demonstration of which microbe in the coculture is responsible for the production of a specific metabolite is required.

It is interesting to note that multiple metabolites were elicited in high abundance using LPS and coculture (Figure 3), being more abundant than those observed for the SNP treatment. This result may be due to the fact that the signals indicated by LPS and coculture are specifically competitive, so the bacteria responds with an increase in the production of essential metabolites to guarantee the colonization of the environment. The effect of the concentration at which metabolites are produced in response to environmental signals has not been studied in-depth but could provide new premises for a more detailed understanding of the expression of bioactive secondary metabolites directly related to competition.

### 3.3. Antibacterial Activity was Dependent on Culture Conditions and All Elicitation Treatments—LPS, SNP, and Coculture—Showed Improved Activity

**Figure 4:**
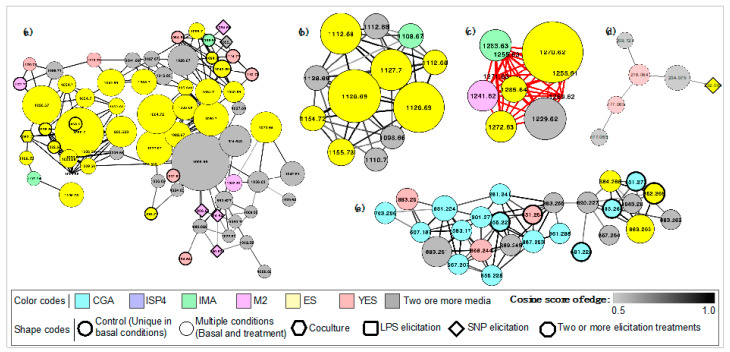
Molecular networking including adduct ions annotated as antibiotics (highlighted in yellow) produced by Antarctic bacteria strains in different culture conditions and obtained by HPLC-QTOF-MS/MS metadata analysis through GNPS (Global Natural Products Social Molecular Networking). Metabolites analogues for the different strains are shown as: (**a**) Surfactins of So1b; (**b**) Massetolides of Dwa41.01b; (**c**) Actinomycin of So13.3; (**d**) Brefeldin A of SoD9b; (**e**) Bacillibactin of So1b. Each spectrum is shown as a node labeled with the monoisotopic mass (*m/z* [M^+^H]^+^), with size proportional to the abundance. Related metabolites are link by edges with cosine scores between 0.5 and 1. Elicitation treatment and culture conditions are represented as shown in the legend based on the color and shape of the node.

The metadata obtained for each strain was compared with the known compounds in the GNPS library, allowing the annotation of some molecules. GNPS uses the DEREPLICATOR algorithm for natural products obtained from metabolomic experiments and includes data annotation using a reference library with approximately 220,000 MS/MS spectra, which facilitate the detection of new compounds [31]. Most of the annotated metabolites corresponded to peptide and lipid precursors commonly found in bacteria such as phosphatidylethanolamine and methoxycinnamic acid, but the annotation of known antibiotic compounds was also noted for 22 of the 34 strains included in this analysis (Table 1). The annotation of these compounds is based on an MQScore greater than 0.7, which suggests a chemical similarity greater than 70% between the compared spectra. These results show the production capacity of antimicrobial compounds of Antarctic bacteria. Some of the annotated compounds were found to be part of molecular interaction networks, which also contain multiple adduct ions with no annotation, produced both in basal conditions and in elicitation treatments (Figure 4). It is possible that many of the unidentified molecules in these clusters correspond to analogues of the annotated putative antibiotics, and represent an opportunity to facilitate the identification and elucidation of chemical structures, as has been reported for the discovery of new antibiotics based on the molecular networking [72,73]

Strains So1b and Dwa41.01b showed networks of related molecules of high monoisotopic mass with high abundance (Figure 4a–b), which indicates that these molecules are produced in greater quantity than the others and therefore their metabolic and physiological role could be crucial for the development of the bacteria. In this case, some members of each network were identified as putative surfactins for So1b and massetolides for Dwa41.01b. The surfactins are lipopeptides described in multiple genera of bacteria (e.g., *Bacillus*, *Streptomyces*, and *Pseudomonas*) with variable biological activities according to their aminopeptide composition and modifications in their fatty acid chain, including antimicrobial, surfactant, and anticancer activity [74]. On the other hand, massetolides are cyclic lipopeptides with reported antimicrobial activity, whose biosynthetic pathway has been described in *Pseudomonas fluorescens* [75,76]. Like the surfactins, structural variants have allowed the detection of multiple analogous compounds of the massetolides, showing the possibility that the strains So1b and Se41.02b are producing multiple variants or analogs of these compounds that are reflected in the identified metabolite network.

Regarding the antibacterial activity, few of the evaluated strains showed inhibition of Gram-negative pathogens (x5) and only the strains Se16.2.3 and Se63.02b maintained this characteristic in multiple evaluated conditions. The main differences between culture conditions were observed in eight of the Antarctic strains evaluated (Table 2), from which it is possible to deduce that the composition of the culture media has an important effect on the observed antibiotic activity and varies depending on the strain. In addition, in all strains, at least in 3 of the 6 assessed media did not generate antibacterial compound production. Elicitation with coculture and with LPS generated positive responses in the inhibitory activity for 4 and 5 strains, respectively, while the addition of SNP improved the activity of 3 strains. The inhibition against *E. faecalis* and *S. aureus* was greater mainly when the cultures were made under the coculture condition (Se18.01b, So1b, So13.3, and Se63.02b) and, in a single case, with the addition of SNP (So64.6b for *S. aureus* and Se63.03b for *E. faecalis*), while for *E. coli* there were no positive responses in these treatments. Nonetheless, two strains (Dwa41.01b and Se63.02b) showed inhibition of *E. coli* dependent of the culture media only when the LPS was present (Table 2). This indicates that the LPS could be a good elicitor for the induction and detection of responses against Gram-negative bacteria.

Our results agree with multiple reports that evidence the variation in the antimicrobial activity of Actinobacteria according to the culture conditions employed [24,25,27,77]. In fact, it is possible that more differences can be found by increasing the amount of extract used. In our work, the extract was not concentrated, and considering that the cultures were performed in microbioreactors (1.5 mL), only the metabolites in high abundance or with strong activity were evidenced. This allowed us to define the most important differences in antibacterial activity. Nevertheless, assays with higher concentrations of crude extract might be useful to reveal subtler variations between culture conditions and the study of the bacteria response to different environmental signaling agents.

Using the metabolomics data, in most cases, it was possible to identify the adduct ions that are produced under the specific conditions of increased antimicrobial activity (Figure 4). In all cases, at least 10 different metabolites were evidenced that could be responsible for the observed activity, thereby, being of interest for its elucidation and its possible analogues. In addition, for the strains that presented antimicrobial activity, it was possible the annotation of multiple putative antibiotic metabolites in 6 strains (Table 1; strains So13.3, Dwa41.01b, Se63.03b, So1b, SoD9b, and Se18.02b). Particularly, in the case of the strain So13.3, it was found that it produces 12 metabolites under the conditions of greatest antimicrobial activity (coculture on IMA and ISP4 culture media), which are also produced under basal conditions with lower intensity (Figure S1). Within these molecules, is the compound noted as a possible Actinomycin D analogue (Figure 4c, metabolite of 1270 *m/z*), which also occurs in higher abundance. This explains the greater antimicrobial activity observed after coculture treatment, since it probably has a higher concentration of antibiotics (i.e., actinomycin analogues) based on the abundance and intensity detected by MS/MS data analysis with at least 1.6 fold change (Figure 4c, Figure S1). This result is also of interest because the compound is in a network with multiple metabolites that are likely to be analogues of actinomycin D (Figure 4c). The future elucidation of its structure is important since, due to its mass (1270 *m/z*), it can be inferred that it could be a methylated analogue of actinomycin D (with a molecular weight of 1255 g/mol) with potential differentiated activity, as with others analogs already reported [78].

### 3.4. Gene Clusters on the Genomes Suggest Antibacterial Potential of Antarctic bacteria and Few of Them Were Associated with Metabolomics Profile and Annotation

The results for each metabolome were used to determine which of the Antarctic strains possess the greatest potential for the discovery of new secondary metabolites, based on the greater diversity of compounds produced in the sum of conditions; greater diversity and possible elicited compounds, with particular interest in high mass molecules and molecular networks between similar molecules; as well as multiple annotated putative antibiotics and antibacterial activity improvement when culture conditions varies. Based on this criteria, strains Dwa41.01b, Se16.2.3, Se63.02b, So1b, So64.6b, So13.3, SoD9b were selected for complete genome sequencing and characterization of genetic clusters associated with secondary metabolite biosynthesis. To achieve this, sequencing was performed using ONT and Illumina technologies by a hybrid assembly approach, which allows the obtention of long reads and high-quality complete assemblies, suitable for the correct detection of repetitive regions frequently found in some biosynthetic gene clusters (BGCs) such as NRPSs [79].

Genome assembly was achieved in a single contig (complete genome) for all the strains, showing lengths from 4.1 to 9.6Mb with high completeness and good quality results (Table 3). Identity was confirmed by BLAST comparison and phylogenetic analysis using the 16S rRNA gene (Figure S2). Results revealed that our Antarctic strains belong to Actinobacteria (*Streptomyces fildesensis* and *Microbacterium* sp.), Proteobacteria (*Sphingomonas alpina*, *Stenotrophomonas maltophilia* and two *Massilia* sp.), and Firmicutes (*Bacillus subtilis*) (Table 3, Figure S2). *Streptomyces fildesensis* was first described and isolated from Fildes Bay, Antarctic [80], and we previously reported its antimicrobial potential and genomic characteristics [81]. *Microbacterium* sp. has been widely reported in the Antarctic and its capacity to produce pigments has been described [82,83]. Additionally, several works have also reported the isolation and antibiotic activity of *Bacillus* strains [84]. On the other hand, *S. maltophilia* is a psychrotolerant bacterium previously isolated from Antarctic samples and reported as a producer of extracellular protease and antifreeze proteins [85,86,87,88]. In the case of *Massilia* sp., it was reported in cryoconites of the Antarctic and therefore studied as organic carbon degradator [89], it has also been described as one of the most representative groups in Antarctic microbial mats [90]. Regarding *Sphingomonas*, a new species of the genus was discovered in 2017, isolated from the Antarctica [91]. However, to the best of our knowledge, any isolates or descriptions of *Sphingomonas alpina* have been made from Antarctic samples, as this species was first described in 2012 from a soil isolation obtained from the Austrian Alps [92].

**Figure 5:**
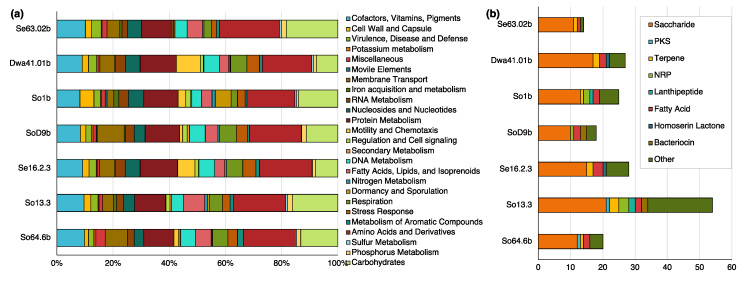
Genomic characteristics of selected Antarctic bacteria strains as potential producers of new antimicrobial secondary metabolites. (**a**) Distribution of coding sequences based on the genome annotation and categorized by feature in subsystems; (**b**) Amount and type of biosynthetic gene clusters identified with AntiSMASH.

Genome annotation showed that these strains have 4495 to 8990 coding sequences, with expected GC contents of 68% to 70% for Actinobacteria, 63% to 66% for Proteobacteria and 44% for Firmicutes (Table 3). Common characteristics of bacteria were found, including a higher abundance of genes related to the production of cofactors, vitamins and pigments; nucleotides and nucleosides; fatty acids, lipids and isoprenes; protein metabolism, membrane transport, and respiration (Figure 5a). Plasmids and other mobile elements were found only in strains So1b, Se16.2.3, and Dwa41.01b. Also, as expected, the strain So1b contains genes related to sporulation and dormancy, since it is the only spore-forming microorganism included in this study. Interestingly, genes associated with cold-adaptation were also identified for all the strains, including the exoribonuclease R, that may have a role in energy saving and is essential at low temperatures as part of degradosome of species like *Pseudomonas syringae* [93,94,95]. Biosynthetic gene clusters producing secondary metabolites (BGCs) varied from 14 for strain Se63.02b to 54 for strain So13.3 (Figure 5b). In general, a higher production of saccharides, terpenes, and fatty acids was found, followed by bacteriocins, NRPS (no-ribosomal peptide synthase), and PKS (polyketide synthase) (Figure 5b, Table S3). This data is in accordance with the current knowledge on bacterial secondary metabolism since it has been reported that 40% of BGCs in prokaryotes are saccharides, which are found in 93% of bacteria and 33% of those represents half of the total BGCs [96]. Specifically, lipopolysaccharides and capsular saccharides have essential roles in interaction between microorganisms and/or with hosts, while diffusible saccharides can have many biological activities—mainly antibacterial—however, the function of most saccharides remains unknown [96,97]. Numbers and types of BGCs were not completely associated with phylogenetic origin of the strains, as it is shown that actinobacterial strains showed both the greatest and the lowest number of BGCs detected in the genomes (Figure 5, Figure S1); as well, closely related *Massilia* sp. strains Se63.02b and Dwa41.01b shares most but not all of the BGCs detected, possibly due to recent gene acquisition events (Figure S1, Table S3). Likewise, a previous report on other bacteria species states that even though BGC distribution could correlate with species phylogeny, the BGC diversity could not be explained by vertical evolution and more data is needed to understand BGC diversification [98].

**Figure 6:**
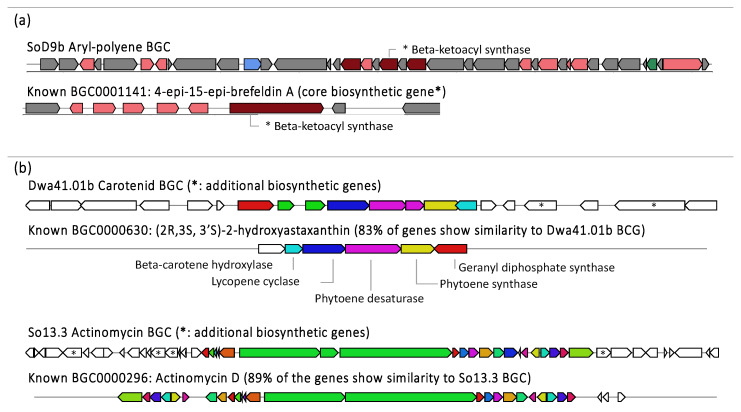
Biosynthetic gene clusters (BGCs) graphics identified in the genomes of different Antarctic bacteria compared with known BGCs of the Repository of Known Biosynthetic Gene Clusters MiBiG. (**a**) BGCs of Aryl-polyene with possible structural similarity to Brefeldin A based on its shared core gene coding for a common type of enzyme. (**b**) BCGs from Antarctic strains with its closest homologous known gene cluster (gene similarity shown in brackets), additional biosynthetic genes found in the BCGs of Antarctic strains are marked with “*”. The metabolomic products of the shown BGCs were also detected and annotated by massive HPLC-QTOF-MS/MS data.

In all the strains, some BGCs showed similarities with known antibiotics, but most of them did not match with any known BGCs (Table S3), which is interesting considering that Antarctic bacteria have been proposed as an unexplored source of new secondary metabolites. BGCs identified in the genome were compared with metabolites detected and annotated by MS/MS. The production of some of these metabolites at specific culture conditions was confirmed. Among them, the bacillibactin-producing cluster of strain So1b—a known siderophore of *Bacillus* [99]—was annotated by metabolomic analyses. This compound was produced in ISP2 when elicited with SNP and GYA culture media with or without elicitation of SNP with a monoisotopic mass of 883.26 *m/z*, while an analogue of 852.3 *m/z* was produced in GYA basal medium or ISP2 in the presence and absence of SNP. In addition, these molecules integrated a molecular network with at least 20 additional molecules (Figure 4e) produced under different conditions, which may represent analogues of this putative bacillibactin. Likewise, a putative brefeldin A-related analogue was detected in extracts from strain SoD9b only when it was cultivated on R2YE medium and elicited with SNP, but related molecules were produced in other media (Figure 4d). Brefeldin A is produced by fungi and has multiple biological activities, including antitumor, antiviral, and antifungal, so its structure has drawn attention as a potential source for the development of new drugs and the search for new analogues [100]. Within the BGCs identified for the genome of SoD9b, no similarities were found with brefeldin A reference cluster. However, an aryl polyene was identified (Figure 6), whose main biosynthetic gene is a beta-ketoacyl synthase and the unique biosynthetic gene coding for core enzymes on Brefeldin A is also a beta-ketoacyl synthase. Structure elucidation and BGCs expression should be achieved to confirm which BGC is responsible for the production of the brefeldin-related molecule; however, based on our actual data it might be possible that the metabolite annotation is associated with the product of this aryl polyene BGC, resulting in a core fatty acid structure related with the MS/MS fragmentation ion pattern of brefeldin A. Consequently, this compound and its analogues could have structural variations of interest for future developments and studies associated with biological activities of brefeldin A-related molecules.

On the other hand, for the strain Dwa41.01b a metabolite was detected and annotated as a putative astaxanthin (596.8 *m/z*), with a monoisotopic mass of 616.5 *m/z*. Astaxanthin is known for its extensive biological activity—generally higher than other carotenoids—including antioxidant, antitumor, antibiotic, and anti-inflammatory [101,102], and has been used for the creation of antimicrobial polymers [103]. In the genome of this Antarctic strain, a carotenoid producing BGC was identified, containing astaxanthin biosynthetic genes such as lycopene cyclase, phytoene synthase, and beta-carotene hydroxylase, but also comprises additional biosynthetic genes (e.g., oxidoreductase and additional dehydrogenases) (Figure 6). This information suggests that this strain produces a carotenoid with structural similarities to astaxanthin, which was obtained in all culture media, with or without SNP; however, the addition of LPS seems to have an inhibitory role in the production of the carotenoid. In the case of the strain So13.3, the presence of the actinomycin BGC was confirmed, as well as its structural match with actinomycin D based on the prediction from the core biosynthetic genes (Figure S3), which are shared with the reference actinomycin D known BGC. However, strain So13.3 contains additional biosynthetic genes (Figure 6) consisting of oxidoreductases, monooxygenase and adenylosuccinate lyase enzymes; this suggests that actinomycin D-related analogues were indeed produced by *S. fildesensis* So13.3 and detected in our GNPS data. Consequently, this BGC is of great interest for the search for possible new actinomycin analogues, whose production is stimulated by the coculture as confirmed by MS/MS and antimicrobial activity assays.

For other strains, there was no direct coincidence of the annotation by comparison of the MS/MS spectrum and the identification of the BGCs in the genome, even though genetic clusters producing NRPS or PKS with similarities to known antibiotics were found (e.g., Se63.02b and Se16.2.3b), to which observed antibiotic activity could be attributed. The lack of coincidences between both annotation approaches (metabolomics and genomics) is mainly due to the fact that not all compounds are found in the databases of both tools (i.e., GNPS for MS/MS data and AntiSMASH for genomic data) and there are few applications linking these types of datasets. It is also likely that not all BGCs were expressed even after the screening of culture conditions and were not detected during MS/MS analysis. Finally, in the case of most new compounds there are still no descriptions or tools allowing the association of chemical massive data (e.g., MS/MS) with genetic information, with the exception of approaches for ribosomal peptide products such as the currently available MetaMiner [104] tool, which improves previous works on peptodogenomics (e.g., RiPPquest, NRPquest, Pep2Path). Efforts are needed to improve our knowledge on secondary metabolism for the development of new functional bio- and chemoinformatics tools in drug discovery, particularly for saccharides, fatty acid, terpenes, PKS, and other non-peptide metabolites more frequently found on microbes. Considering this, there is a current importance in using systemic searches of metabolites (as included in this work) for prioritizing BGCs of interest prior to experimental characterization [96,105].

## 4. Conclusions

One bacterial strain is capable of producing a broad range of bioactive metabolites, including various analogs with slight but functional modification of core structures. Nevertheless, those secondary metabolites are not directly involved in growth or reproduction, thus the biosynthesis pathways to produce them will be only activated if a real necessity is sensed in the environment. It is not completely understood how bacteria tune their response to environmental changes in a cost-effective way, but it is well-known that these processes have important ecological roles in interactions with other organisms, signaling, and defense.

Activation of BGCs for discovery of new antibiotics has been reported; however, among the multiple methods, none have shown promising results for broad range application. Our work confirmed the favorable elicitation of metabolites of Antarctic bacteria by the simulation of environmental threats during culture, including the addition of LPS, SNP—nitric oxide donor—and coculture with their natural cohabitants and potential competitors. Based on our results, it is possible to recommend those strategies for the activation of biosynthetic pathways as potent pleiotropic regulators in the global bacterial response. Nonetheless, as expected, the response varies considerably from one bacterium to another. In particular, the enhancement of antibacterial activity could be achieved only by testing multiple conditions, with virtually non-common patterns to define a suitable and unique treatment for the activation of bioactive compounds.

We highlight the necessity of including screening elicitation conditions for bioactivity evaluation of untapped bacteria to exploit the vast diversity of metabolites they harbor, since such production will be promoted by the environment they are facing. Moreover, adequate nutritional conditions are a key factor for the biosynthesis of metabolites, therefore, it is essential to attempt the growth of bacteria under multiple nutritional profiles along with elicitors exposure. In this context metabolomics based on MS/MS data and recent analysis tools (i.e., MS networking) are important approaches in high-throughput screening, allowing the characterization and prioritization of natural sources for an accelerated drug discovery. Advances on culture-dependent and culture-independent methods, including integrative approaches for comparison of multi-omics data and precise predictions of metabolites from complex ecosystems, promise to break into a new era in antimicrobial research and development.

## Figures and Tables

**Table 1:** Annotation of metabolites with similarity to antibiotics on the GNPS public library (MQScore > 0.70) based on HPLC-QTOF-MS/MS metadata from Antarctic bacteria strains.

Strain ID	Annotated Antibiotics
So13b	Aphidicolin
Dwa41.01b*	Sarmentoside, Massetolides
Se14.01b	Aphidicolin
Se16.2.5	Monolinolein, Tetramisole
Se18.01b*	Anisomycin, Aphidicolin, Sarmentoside
Se32.01b	Aphidicolin, Anisomycin
Se41.02b	Sarmentoside, Massetolides, Viscosin
Se5.01b	Monoelaidin
Se5.02b	Monoesin, Monoelaidin, Sarmentoside, Monoactin, Dinactin
Se63.02b*	Monoelaidin, Sarmentoside, Aphidicolin, Antimycin
So1b*	Surfactin, Sarmentoside, Dactinomycin, Bacillibactin, Actinomycin
So10b	Dinactin, Sarmentoside
So13.3*	Streptorubin, Surfactin, Sarmentoside, Prenyletin, Dactinomycin, Actinomycin, Piericidin
So16.17	Surfactin
So1d	Xefoampeptide, Monoelaidin, Sarmentoside, Enterobactin.
So2b	Monoelaidin
So5b	Sarmentoside, Anisomycin
So64.3b	Surfactin, Sarmentoside, Dinactin
So6b	Monoactin, Dinactin, Antimycin, Piericidin
So9b	Sarmentoside
SoD9b*	Monoelaidin, Brefeldin A
Wa41.01b	Bassianolide, Sarmentoside

*: Strains with antibacterial activity confirmed by disk diffusion assay using its crude extract.

**Table 2:** Antibacterial activity of Antarctic bacterial strains cultivated under different conditions and elicitation treatments, determined by inhibition zones (mm) in disk diffusion assay against the pathogenic bacteria *Staphylococcus aureus, Escherichia coli* and *Enterococcus faecalis.*

Antarctic Strain	Untreated						LPS Elicitation						SNP Elicitation						Coculture						Pathogen
	M2	IMA	ISP4	YES	CGA	ES	M2	IMA	ISP4	YES	CGA	ES	M2	IMA	ISP4	YES	CGA	ES	M2	IMA	ISP4	YES	CGA	ES	
**Se18.01**	-	-	-	-	-	-	-	-	-	-	-	-	-	-	-	-	-	-	-	-	**6.5**	-	**9**	-	***S. aureus***
	-	-	-	-	-	-	-	-	-	-	-	-	-	-	-	-	-	-	-	-	-	-	-	-	***E. coli***
	-	-	-	-	-	-	-	-	-	-	-	-	-	-	-	-	-	-	-	-	-	-	-	-	***E. faecalis***
**Se16.2.3**	-	-	-	-	-	-	-	-	-	-	-	-	-	-	-	-	-	-	-	-	-	-	-	-	***S. aureus***
	-	-	-	**7**	-	-	**6.5**	-	-	-	-	-	**6.5**	-	-	-	**6.5**	-	-	-	-	-	-	-	***E. coli***
	-	-	-	-	-	-	-	-	-	-	-	-	-	-	-	-	-	-	-	-	-	-	-	-	***E. faecalis***
**So1**	**6.5**	-	-	**6.5**	-	-	-	-	-	-	-	-	-	-	-	-	-	-	-	**14**	**11**	-	-	-	***S. aureus***
	-	-	-	**6.5**	-	-	-	-	-	-	-	-	-	-	-	-	-	-	-	-	-	-	-	-	***E. coli***
	**6.5**	-	-	**7.5**	-	-		-	-	-	-	-	-	-	-	-	-	-	-	**18**	**11**	-	-	-	***E. faecalis***
**SoD9**	-	**12**	-	-	-	-	-	**12**	-	-	-	-	-	-	-	-	-	-	-	-	-	-	-	-	***S. aureus***
	-	-	-	-	-	-	-	-	-	-	-	-	-	-	-	-	-	-	-	-	-	-	-	-	***E. coli***
	-	-	-	-	-	-	-	-	-	-	-	-	-	-	-	-	-	-	-	-	-	-	-	-	***E. faecalis***
**So64.6**	-	-	-	-	-	-	-	-	**7**	-	-	-	-	-	**8**	-	-	-	-	-	-	-	-	-	***S. aureus***
	-	-	-	-	-	-	-	-	-	-	-	-	-	-	-	-	-	-	-	-	-	-	-	-	***E. coli***
	-	-	-	-	-	-	-	-	-	-	-	-	-	-	-	-	-	-	-	-	-	-	-	-	***E. faecalis***
**So13.3**	-	**11**	**7**	-	-	-	-	-	-	-	-	-	-	-	-	-	-	-	-	**14**	**11**	-	-	-	***S. aureus***
	-	-	-	**7**	-	-	**6.5**	-	-	-	**6.5**	-	-	-	-	-	-	-	-	-	-	-	-	-	***E. coli***
	-	-	-	-	-	-	-	**12**	-	**7**	**6.5**	-	-	-	-	-	-	-	-	**18**	**11**	-	-	-	***E. faecalis***
**Se63.02**	-	-	-	-	-	-	-	-	-	-	-	-	-	-	-	-	-	-	-	-	**6.5**	-	**9**	-	***S. aureus***
	-	-	-	**7**	-	-	**6.5**	-	-	**6.5**	**7**	-	**6.5**	-	-	-	-	-	-	-	-	-	-	-	***E. coli***
	-	-	-	-	-	-	**8**	-	-	-	-	-	**9.5**	-	-	-	**7**	-	-	-	-	-	-	-	***E. faecalis***
**Dwa41.01**	**6.5**	**6.5**	-	-	-	-	-	-	-	-	-	-	**7**	**9**	-	-	-	-	-	-	-	-	-	-	***S. aureus***
	-	-	-	-	-	-	**6.5**	-	-		-	-	-	-	-	-	-	-	-	-	-	-	-	-	***E. coli***
	-	-	-	-	-	-	-	-	-	-	-	-	-	-	-	-	-	-	-	-	-	-	-	-	***E. faecalis***

-: No inhibition detected. Color matches values of: 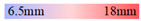.

**Table 3:** Quality parameters and general genomic data of assembled genomes.

Strain ID	Closest Taxa (Identity %)*	Completeness	Length	Contigs	Coverage	Plasmids	GC Content (%)	Number of CDSs	Number of ARNs
So13.3	*Streptomyces fildesensis (100%)*	100.00%	9.563.913	1	140	0	70.4	8990	87
So64.6b	*Sphingomonas alpina (99.6%)*	99.66%	5.570.175	1	150	0	63.5	4922	51
SoD9b	*Stenotrophomonas maltophilia (99.8%)*	98.64%	4.415.649	1	205	0	66.8	4495	83
So1b	*Bacillus subtilis (100%)*	100.00%	4.070.574	1	220	0	43.9	4213	116
Dwa41.01b	*Uncultured bacterium – Massilia sp. (98.3%)*	96.68%	5.348.447	1	185	1	65.2	5456	90
Se63.02b	*Microbacterium sp. (99.8%)*	93.33%	4.082.358	1	250	0	68.5	4650	51
Se16.2.3	*Massilia sp. (98.3%)*	95.13%	5.349.291	1	225	1	65.3	5446	90

*: Based on comparison of the gene 16S rRNA with BLASTN. CDSs: Coding Sequences.

## Supplementary Materials

The following are available online at https://www.mdpi.com/2218-273X/10/5/673/s1, Table S1: Origin data of Antarctic bacterial strains included in this study, Table S2: Composition of culture media used in this study, Table S3: Biosynthetic gene clusters (BGCs) identified on the genomes of selected Antarctic bacteria strains as potential source of novel antimicrobial compounds, Figure S1: HPLC-MS/MS spectra of organic crude extracts from the Antarctic strain Streptomyces fildesensis So13.3 under different culture conditions: (a) IMA medium with no elicitation treatment (basal condition) and (b) IMA medium with coculture elicitation strains So13.3 + So1b. Peak correspondent to putative Actinomycin D is showed and labeled with the intensity of this compound detected for each culture condition, Figure S2: Maximum-likelihood phylogenetic tree based on complete 16S rRNA gene sequences showing the genetic distances between Antarctic bacterial strains selected in this study and closely related species based on 16S rRNA reference sequences from DDBJ/ENA/GenBank database, Figure S3: Chemical structures predicted for biosynthetic gene cluster similar to Actinomycin D from the Antarctic strain So13.3 genome (Scaffolds 1-3) compared to the structure of the known drug. The predicted structures were based on the core biosynthetic genes using PRISM 4 (http://grid.adapsyn.com/prism/#!/prism).
