# Supplementary material for: Genomic and Metabolomic Analysis of Antarctic Bacteria Revealed Culture and Elicitation Conditions for the Production of Antimicrobial Compounds
Source: Biomolecules. 2020 Apr 27;10(5):673. doi: 10.3390/biom10050673 (PMC7277857; doi:10.3390/biom10050673)
Supplement: Supplementary file 1 [file biomolecules-10-00673-s001.pdf]

biomolecules

MDPI

Supplementary Materials

Table S1. Origin data of Antarctic bacterial strains included in this study.

Strain ID*	Sampling Location	Sampling Date	Strain in Coculture
So1b	Fildes Bay (a)	2013/2014	So13.3
So13.3	Fildes Bay (a)	2013/2014	So1b
So64.6b	Ardley Island (ASPA 150)	2013/2014	So32D
So5b	Collins Glacier beach area (ASPA 125)	2013/2014	So27D
Se14.01b	Armonía point (b) (ASPA 133)	2013/2014	Se41.02b
Se5.02b	Hannah point, Livingston Island (c) (ASPA 126)	2013/2014	Se5.01b
Se18.01b	Fildes Bay (a)	2013/2014	Se63.02b
Se28.01b	Dee-Greenwich Island (a)	2013/2014	Se32.01b
Se32.01b	Dee-Greenwich Island (a)	2013/2014	Se28.01b
Se41.02b	Armonía point (ASPA 133)	2013/2014	Se14.01b
Wa41.01b	Armonía point (ASPA 133)	2013/2014	Dwa41.01b
So64.3b	Ardley Island (ASPA 150)	2013/2014	So64.6b
So27D	Collins Glacier beach area (ASPA 125)	2013/2014	So5b
So10b	Collins Glacier beach area (ASPA 125)	2013/2014	So2b
So2b	Collins Glacier beach area (ASPA 125)	2013/2014	So10b
So9b	Collins Glacier beach area (ASPA 125)	2013/2014	So6b
Dwa41.01b	Fildes Bay (a)	2013/2014	Wa41.01
Se4.02b	Hannah point, Livingston Island (ASPA 126)	2013/2014	Se5.02b
So11b	Collins Glacier beach area (ASPA 125)	2013/2014	SoD9b
Se63.02b	Fildes Bay (a)	2013/2014	Se18.01
So1d	Collins Glacier beach area (ASPA 125)	2013/2014	So5b
So13b	Fildes Bay (a)	2016	So02b
So3.2b	Robert Island (ASPA 112)	2016	Se32.02b
Se32.02b	Robert Island (ASPA 112)	2013/2014	So3.2b
Se5.01b	Hannah point, Livingston Island (ASPA 126)	2013/2014	Se5.02b
So6b	Collins Glacier beach area (ASPA 125)	2013/2014	So9b
SoD9b	Collins Glacier beach area (ASPA 125)	2013/2014	So11b
So02b	Fildes Bay (a)	2016	So13b
Se16.2.2	Collins Glacier beach area (ASPA 125)	2016	Se16.2.3
Se16.2.3	Collins Glacier beach area (ASPA 125)	2016	Se16.2.2
Se16.2.5	Collins Glacier beach area (ASPA 125)	2016	Se16.2.3
So16.8	Armonía point (ASPA 133)	2016	So16.17
So16.17	Armonía point (ASPA 133)	2016	So16.8
So16.14.3	Robert Island (ASPA 112)	2016	So16.14.1

*Name codes *So*: soil, *Se*: sediment, *Wa*: water, *Dwa:* 10 m deep water, .*ASPA:* Antarctic Specially Protected Area number. (a) Area with human presence. (b) Lake sediment 0.3 m deep. (c) Marine sediment 0.3 m deep.

Table S2. Composition of culture media used in this study.

Media ID	Composition Per Liter
**M1**	Peptone (2.0 g)<br>Yeast extract (4.0 g)<br>Starch (1 0.0 g)
**ISP2**	Yeast extract (4.0 g)<br>Malt extract (10.0 g)<br>Glucose (4.0 g)
**M2**	Mannitol (40.0 g)<br>Maltose (40.0 g)<br>Yeast extract (10.0 g)<br>K2HPO4 (2.0 g)<br>MgSO4.7H2O (0.5 g)<br>FeSO4.7H2O (0.01 g)
**SCA**	Starch (10.0 g)<br>Casein (0.3 g)<br>KNO_3 (2.0 g)<br>NaCl (2.0 g)<br>K_2HPO_4 (2.0 g)<br>$MgSO_4.7H_2O$ (0.05 g)<br>$CaCO_3$ (0.02 g)

	$FeSO_4.7H_2O$ (0.01 g)
**IMA**	Yeast extract (4g)<br>Malt extract (10g)<br>Glucose (4g)<br>Mannitol (40g)
**SDB**	Peptidic digest of animal tissue (5.0 g),<br>Casein pancreatin disgest (5.0 g),<br>Dextrose (40.0 g)
**CCA**	Glicerol (30g)<br>Peptone (2g)<br>K_2HPO_4 (1g)<br>NaCl (1g)<br>$MgSO_4.7H_2O$ (0.5g)<br>Trace solution * (5mL)<br>***Trace solution**<br>$CaCl_2x2H_2O$ (3g)<br>$MnSO_4$ (0.2g)<br>$ZnCl_2$ (0.1g)<br>$CuSO4.5H_2O$ (0.025g)<br>$Na_2B_4O_7x10H_2O$ (0.02g)<br>$CoCl_2$ (0.004g)<br>$(NH_4)_6Mo_7O_{24}$ $4H_2O$ (0.01g)
**YEME**	Yeast extract (3g)<br>Peptone (5g)<br>Malt extract (3g)<br>Glucose (10g)<br>Sucrose (170g)
**GYA**	Yeast extract (4g)<br>Malt extract (10g)<br>Glucose (4g)<br>$CaCO_3$ (2g)<br>Starch (20g)
**YES**	Sucrose (150 g)<br>Yeast extract (20 g)<br>MgSO4x7H2O (0.5 g)<br>ZnSO4x7H2O (0.01 g)<br>CuSO4x5H2O (0.005 g)
**ISP4**	Starch (10.0 g)<br>CaCO3 (2.0 g)<br>(NH4)2SO4 (2.0 g)<br>K2HPO4 (1.0 g)<br>MgSO4.7H2O (1.0 g)<br>NaCl (1.0 g)<br>FeSO4.7H2O (1 mg)<br>MnCl2.7H2O (1.0 mg)<br>ZnSO4.7H2O (1.0 mg)
**ES**	100g of Antarctic soil (1 hour agitation prior filtering)
**R2YE**	[1]

[1] Ghimire, G. P.; Koirala, N.; Sohng, J. K. Activation of cryptic hop genes from streptomyces peucetius ATCC 27952 involved in hopanoid biosynthesis. *J. Microbiol. Biotechnol.* **2015**, doi:10.4014/jmb.1408.08058.

Table S3. Biosynthetic gene clusters (BGCs) identified on the genomes of selected Antarctic bacteria strains as potential source of novel antimicrobial compounds.

Dwa41.01b					
**Region**	**Type**	**From**	**To**	**Most similar known cluster**	**Similarity**
**Region 1**	ladderane,fatty_acid	75,852	118,134	pseudomonas quinolone signal	27%
**Region 2**	saccharide	271,126	294,546		
**Region 3**	saccharide	364,464	380,12		
**Region 4**	saccharide	1,126,731	1,151,389		
**Region 5**	saccharide	1,404,293	1,426,859		
**Region 6**	halogenated	1,466,276	1,491,000	WAP-8294A2	30%
**Region 7**	saccharide	1,500,477	1,535,607		
**Region 8**	saccharide	1,545,784	1,566,616		
**Region 9**	saccharide	1,706,299	1,752,277	exopolysaccharide	20%
**Region 10**	saccharide	2,181,871	2,200,672		
**Region 11**	saccharide	2,498,222	2,537,719		
**Region 12**	terpene	2,585,329	2,604,912		
**Region 13**	acyl_amino_acids	2,666,701	2,726,921		
**Region 14**	arylpolyene	3,204,781	3,248,883	APE Vf	35%
**Region 15**	hserlactone	3,275,936	3,296,523		
**Region 16**	fatty_acid	3,375,525	3,398,099		
**Region 17**	saccharide	3,584,363	3,622,907	K53 capsular polysaccharide	15%
**Region 18**	saccharide	3,827,884	3,872,531		
**Region 19**	saccharide	4,186,168	4,207,395	lipopolysaccharide	5%
**Region 20**	terpene	4,301,655	4,325,256	carotenoid	100%
**Region 21**	saccharide	4,346,330	4,403,382		
**Region 22**	saccharide	4,479,885	4,530,756	lipopolysaccharide	14%
**Region 23**	saccharide	4,615,542	4,639,123	O-antigen	14%
**Region 24**	saccharide	4,812,304	4,836,129	lipopolysaccharide	5%
**Region 25**	saccharide	4,840,049	4,860,600		
**Region 26**	acyl_amino_acids,sacc	5,179,912	5,267,121	K53 capsular polysaccharide	10%
**Region 27**	fatty_acid	5,268,349	5,289,470	polyhydroxyalkanoate	50%
**So1b**					
**Region**	**Type**	**From**	**To**	**Most similar known cluster**	**Similarity**
**Region 1**	saccharide,T3PKS	333,107	430,53	dipeptide aldehydes	11%
**Region 2**	saccharide,terpene	435,276	473,443		
**Region 3**	NRPS,betalactone	536,424	618,887	fengycin	100%
**Region 4**	saccharide	665,202	685,619		
**Region 5**	transAT-PKS-	742,139	842,379	bacillaene	**Similarity**
**Region 6**	saccharide	1,006,994	1,027,472		
**Region 7**	saccharide	1,334,496	1,356,064		
**Region 8**	fatty_acid	1,385,610	1,406,452	chejuenolide A / chejuenolide B	11%
**Region 9**	terpene	1,433,936	1,454,715		
**Region 10**	saccharide	1,468,497	1,485,149	zwittermicin A	18%
**Region 11**	fatty_acid	1,501,606	1,522,487		
**Region 12**	halogenated	1,705,352	1,725,585		
**Region 13**	saccharide	1,785,201	1,807,273		
**Region 14**	NRPS	2,137,424	2,201,286	surfactin	78%
**Region 15**	saccharide	2,219,940	2,262,274		
**Region 16**	lanthipeptide	2,332,643	2,357,099	subtilomycin	100%
**Region 17**	saccharide	2,485,752	2,506,785		
**Region 18**	saccharide	2,758,084	2,778,117	bacillomycin D	20%
**Region 19**	saccharide	2,806,497	2,826,120		
**Region 20**	saccharide,other	2,852,963	2,910,931	bacilysin	100%
**Region 21**	sactipeptide,head_to_t	2,917,688	2,939,298	subtilosin A	100%
**Region 22**	saccharide	3,072,095	3,126,189	teichuronic acid	100%
**Region 23**	saccharide	3,194,906	3,256,035	K53 capsular polysaccharide	15%
**Region 24**	saccharide	3,277,735	3,303,013		
**Region 25**	NRPS	3,458,208	3,507,248	bacillibactin	100%
**Region 26**	saccharide	3,532,514	3,563,347		
**Region 27**	saccharide	3,587,706	3,621,555	scytophycin	11%
**Region 28**	saccharide	3,666,012	3,716,386		

**Region 29**	saccharide	3,915,985	3,956,517		
			**Se16.2.3**		
**Region**	**Type**	**From**	**To**	**Most similar known cluster**	**Similarity**
**Region 1**	saccharide	61,393	85,833	O-antigen	9%
**Region 2**	saccharide	258,489	282,201	lipopolysaccharide	5%
**Region 3**	saccharide	284,796	306,672	pyoverdin	1%
**Region 4**	acyl_amino_acids,	626,909	711,754	K53 capsular polysaccharide	10%
**Region 5**	fatty_acid	716,652	734,902	polyhydroxyalkanoate	50%
**Region 6**	fatty_acid	882,123	909,289	pseudomonas quinolone signal	27%
**Region 7**	saccharide	1,066,648	1,089,120		
**Region 8**	saccharide	1,159,045	1,174,700		
**Region 9**	saccharide	1,922,274	1,946,138		
**Region 10**	halogenated	2,261,080	2,285,942	WAP-8294A2	30%
**Region 11**	saccharide	2,501,139	2,547,133	exopolysaccharide	20%
**Region 12**	saccharide	2,674,663	2,695,176		
**Region 13**	saccharide	2,973,950	2,996,371		
**Region 14**	saccharide	3,293,374	3,332,872		
**Region 15**	terpene	3,380,530	3,400,071		
**Region 16**	acyl_amino_acids	3,461,782	3,522,558		
**Region 17**	arylpolyene	4,000,001	4,043,292	APE Vf	40%
**Region 18**	hserlactone	4,071,169	4,091,756		
**Region 19**	fatty_acid	4,170,318	4,194,832		
**Region 20**	saccharide	4,379,674	4,419,034	K53 capsular polysaccharide	15%
**Region 21**	saccharide	4,623,223	4,667,880		
**Region 22**	saccharide	4,981,635	5,002,858	lipopolysaccharide	5%
**Region 23**	terpene	5,097,337	5,120,843	carotenoid	83%
**Region 24**	saccharide	5,142,147	5,198,880		
**Region 25**	saccharide	5,274,999	5,326,287	lipopolysaccharide	17%
			**SoD9b**		
**Region**	**Type**	**From**	**To**	**Most similar known cluster**	**Similarity**
**Region 1**	fatty_acid,saccharide	202,78	235,29	eicoseicosapentaenoic acid	15%
**Region 2**	fatty_acid	525,312	546,261		
**Region 3**	saccharide	569,733	626,198	lipopolysaccharide	54%
**Region 4**	saccharide	661,028	703,576		
**Region 5**	saccharide	727,413	749,236		
**Region 6**	fatty_acid	1,011,227	1,036,777		
**Region 7**	saccharide	1,138,471	1,168,349		
**Region 8**	bacteriocin	1,190,247	1,198,754		
**Region 9**	bacteriocin	1,497,254	1,508,128		
**Region 10**	saccharide	1,876,199	1,898,314	O-antigen	14%
**Region 11**	halogenated	2,292,133	2,311,229		
**Region 12**	saccharide	2,459,986	2,490,582	pseudopyronine A /	18%
**Region 13**	NRPS	2,503,908	2,550,336	streptobactin	23%
**Region 14**	saccharide	3,644,796	3,664,219		
**Region 15**	saccharide	3,853,356	3,881,455		
**Region 16**	saccharide	3,885,147	3,905,260		
**Region 17**	arylpolyene,halogenat	4,222,695	4,266,297	APE Vf	35%
**Region 18**	saccharide	4,286,303	4,308,080		
			**So63.02b**		
**Region**	**Type**	**From**	**To**	**Most similar known cluster**	**Similarity**
**Region 1**	saccharide	521,771	553,102		
**Region 2**	saccharide	644,353	690,098	kosinostatin	3%
**Region 3**	saccharide	1,026,342	1,063,007		
**Region 4**	saccharide	1,114,870	1,154,348		
**Region 5**	saccharide	1,488,720	1,563,242	phosphonoglycans	9%
**Region 6**	saccharide	1,926,695	1,951,760		
**Region 7**	fatty_acid	2,209,409	2,232,061		
**Region 8**	saccharide,betalactone	2,448,883	2,512,420	microansamycin	7%
**Region 9**	saccharide	2,527,091	2,550,083		
**Region 10**	saccharide	2,699,537	2,732,407		
**Region 11**	terpene	2,914,074	2,934,982	carotenoid	28%
**Region 12**	saccharide	3,222,694	3,245,310		

**Region 13**	saccharide	3,426,715	3,445,479		
**Region 14**	saccharide	3,473,784	3,494,764		
			**So64.6b**		
**Region**	**Type**	**From**	**To**	**Most similar known cluster**	**Similarity**
**Region 1**	saccharide	402,957	428,867		
**Region 2**	saccharide	666,331	689,678		
**Region 3**	saccharide	717,381	736,371		
**Region 4**	saccharide	833,072	877,204	glycopeptidolipid	5%
**Region 5**	saccharide	883,546	910,762		
**Region 6**	saccharide	983,304	1,024,979	nogabecin	8%
**Region 7**	lassopeptide,hserlacto	1,448,221	1,470,598		
**Region 8**	fatty_acid	1,532,944	1,561,119		
**Region 9**	saccharide	1,842,988	1,871,436		
**Region 10**	saccharide	2,255,557	2,285,856	O&K-antigen	4%
**Region 11**	halogenated	2,349,570	2,368,140		
**Region 12**	saccharide	2,549,397	2,570,096	lipopolysaccharide	5%
**Region 13**	saccharide	2,722,855	2,745,868	gellan polysaccharide	7%
**Region 14**	saccharide,lassopeptid	3,440,244	3,499,720		
**Region 15**	T3PKS	3,889,391	3,930,452		
**Region 16**	terpene	4,072,179	4,096,869	carotenoid	30%
**Region 17**	fatty_acid	4,115,483	4,136,445		
**Region 18**	saccharide	4,154,895	4,177,593		
**Region 19**	halogenated	4,231,676	4,252,788		
**Region 20**	saccharide	4,417,107	4,453,510		
			**So13.3**		
**Region**	**Type**	**From**	**To**	**Most similar known cluster**	**Similarity**
**Region 1**	lanthipeptide	68,939	91,332	SapB	100%
**Region 2**	fatty_acid	181,707	201,626	actinomycin D	10%
**Region 3**	T3PKS	366,627	406,159	A-47934	23%
**Region 4**	NRPS,other	460,304	527,487	actinomycin D	89%
**Region 5**	CDPS,T3PKS	913,307	960,433	alkylresorcinol	100%
**Region 6**	lassopeptide	1,067,644	1,090,272	clavulanic acid	8%
**Region 7**	NRPS	1,403,665	1,453,533	deimino-antipain	66%
**Region 8**	terpene	1,517,889	1,534,894	ebelactone	5%
**Region 9**	fatty_acid,T2PKS,NRP	1,561,713	1,657,640	spore pigment	83%
**Region 10**	saccharide,terpene	1,799,022	1,834,067	pristinol	100%
**Region 11**	NRPS-	1,855,463	1,902,275	marineosin A / marineosin B	86%
**Region 12**	saccharide	2,110,127	2,134,781		
**Region 13**	saccharide	2,279,201	2,299,855	daptomycin	10%
**Region 14**	saccharide	2,382,299	2,405,253	steffimycin D	5%
**Region 15**	ectoine,saccharide	2,924,964	2,969,079	ectoine	100%
**Region 16**	saccharide	3,110,074	3,133,370		
**Region 17**	saccharide	3,256,328	3,306,690	acarviostatin I03 / acarviostatin	29%
**Region 18**	saccharide	3,318,888	3,351,341	glycopeptidolipid	5%
**Region 19**	fatty_acid	3,574,310	3,595,405		
**Region 20**	siderophore	3,643,627	3,657,431		
**Region 21**	saccharide	3,753,455	3,778,710		
**Region 22**	saccharide	4,115,700	4,140,963		
**Region 23**	saccharide	4,296,616	4,316,343		
**Region 24**	saccharide	4,469,278	4,496,601	cathomycin	20%
**Region 25**	saccharide	4,690,571	4,715,758	neocarzinostatin	6%
**Region 26**	saccharide	4,917,327	4,950,799		
**Region 27**	melanin,saccharide	5,141,629	5,170,222	melanin	100%
**Region 28**	halogenated	5,542,357	5,562,337		
**Region 29**	saccharide	5,618,194	5,657,873	TP-1161	41%
**Region 30**	saccharide	5,886,722	5,928,571	napyradiomycin A80915C	9%
**Region 31**	saccharide	6,092,627	6,116,746		
**Region 32**	T1PKS,fatty_acid,NRP	6,242,873	6,377,035	macrotermycins	46%
**Region 33**	saccharide	6,508,813	6,527,699		
**Region 34**	saccharide,other,halog	6,531,244	6,581,567	A-503083 A / A-503083 B / A-	3%
**Region 35**	siderophore	6,943,207	6,957,918	ficellomycin	3%
**Region 36**	NRPS	7,092,437	7,137,408	enduracidin	10%

**Region 37**	bacteriocin	7,142,716	7,153,177		
**Region 38**	saccharide	7,155,120	7,188,902	ashimides	12%
**Region 39**	saccharide	7,368,836	7,390,778	belactosin A / belactosin C	20%
**Region 40**	terpene	7,486,846	7,510,747	hopene	76%
**Region 41**	NRPS,fatty_acid	7,755,098	7,815,348	myxothiazol	28%
**Region 42**	terpene	7,922,434	7,942,364	nenestatin	3%
**Region 43**	lanthipeptide,terpene	7,942,457	7,984,662	geosmin	100%
**Region 44**	saccharide	8,017,247	8,057,061		
**Region 45**	T1PKS,LAP	8,059,656	8,109,323	maduropeptin	3%
**Region 46**	NRPS,fatty_acid	8,118,137	8,162,078	diisonitrile antibiotic SF2768	61%
**Region 47**	T1PKS,T2PKS,fatty_ac	8,241,505	8,323,840	lysolipin I	65%
**Region 48**	NRPS-like	8,454,616	8,497,189	borrelidin	5%
**Region 49**	bacteriocin	8,508,335	8,520,242		
**Region 50**	melanin	8,793,470	8,803,892	melanin	28%
**Region 51**	NRPS	9,104,379	9,167,226	qinichelins	44%
**Region 52**	saccharide	9,250,594	9,271,013	cosmomycin C	3%
**Region 53**	lanthipeptide	9,293,335	9,315,935	polyoxin A / polyoxin H	5%
**Region 54**	saccharide	9,436,178	9,469,306	acarviostatin I03 / acarviostatin	18%

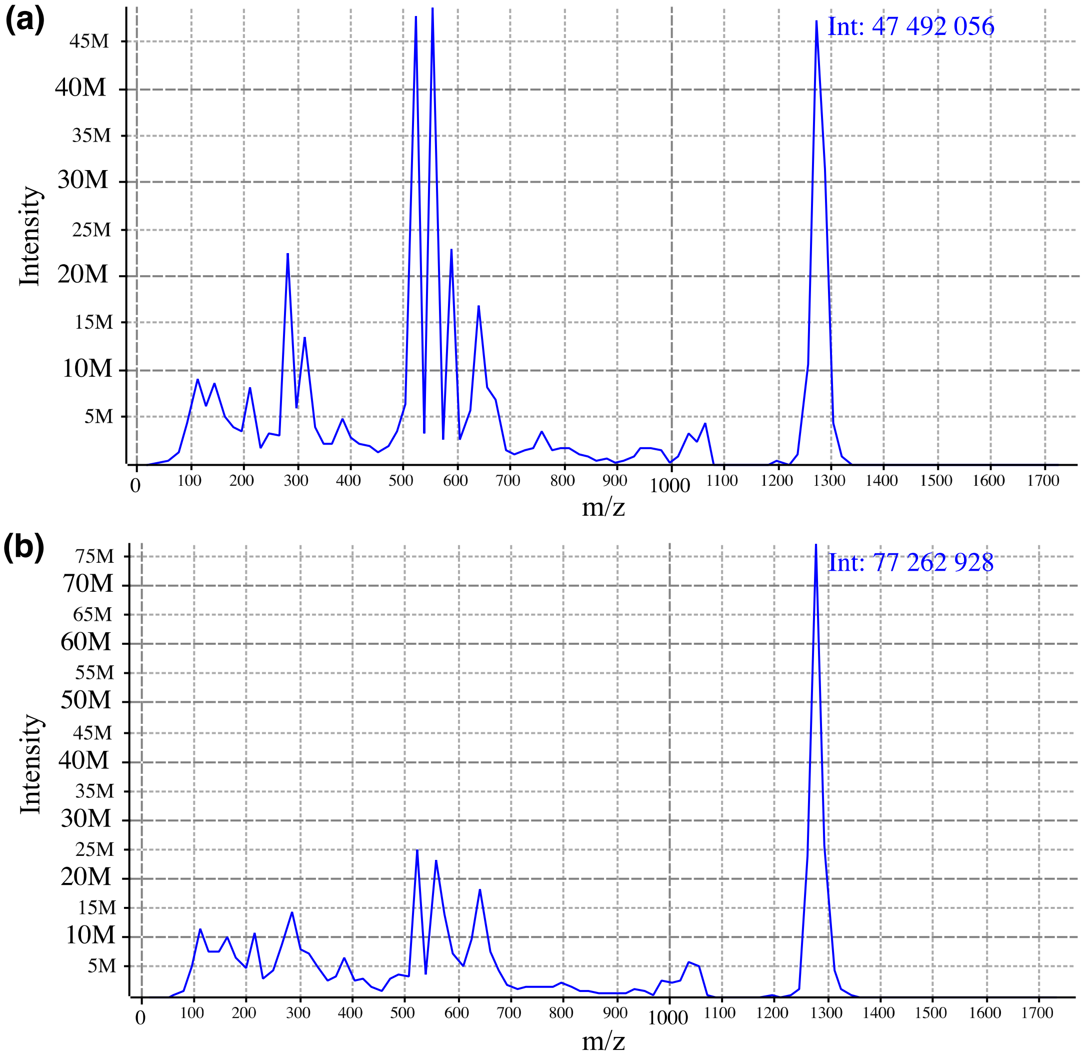

Figure S1. HPLC-MS/MS spectra of organic crude extracts from the Antarctic strain *Streptomyces fildesensis* So13.3 under different culture conditions: (**a**) IMA medium with no elicitation treatment (basal condition) and (**b**) IMA medium with coculture elicitation strains So13.3 + So1b. Peak correspondent to putative Actinomycin D is showed and labeled with the intensity of this compound detected for each culture condition.

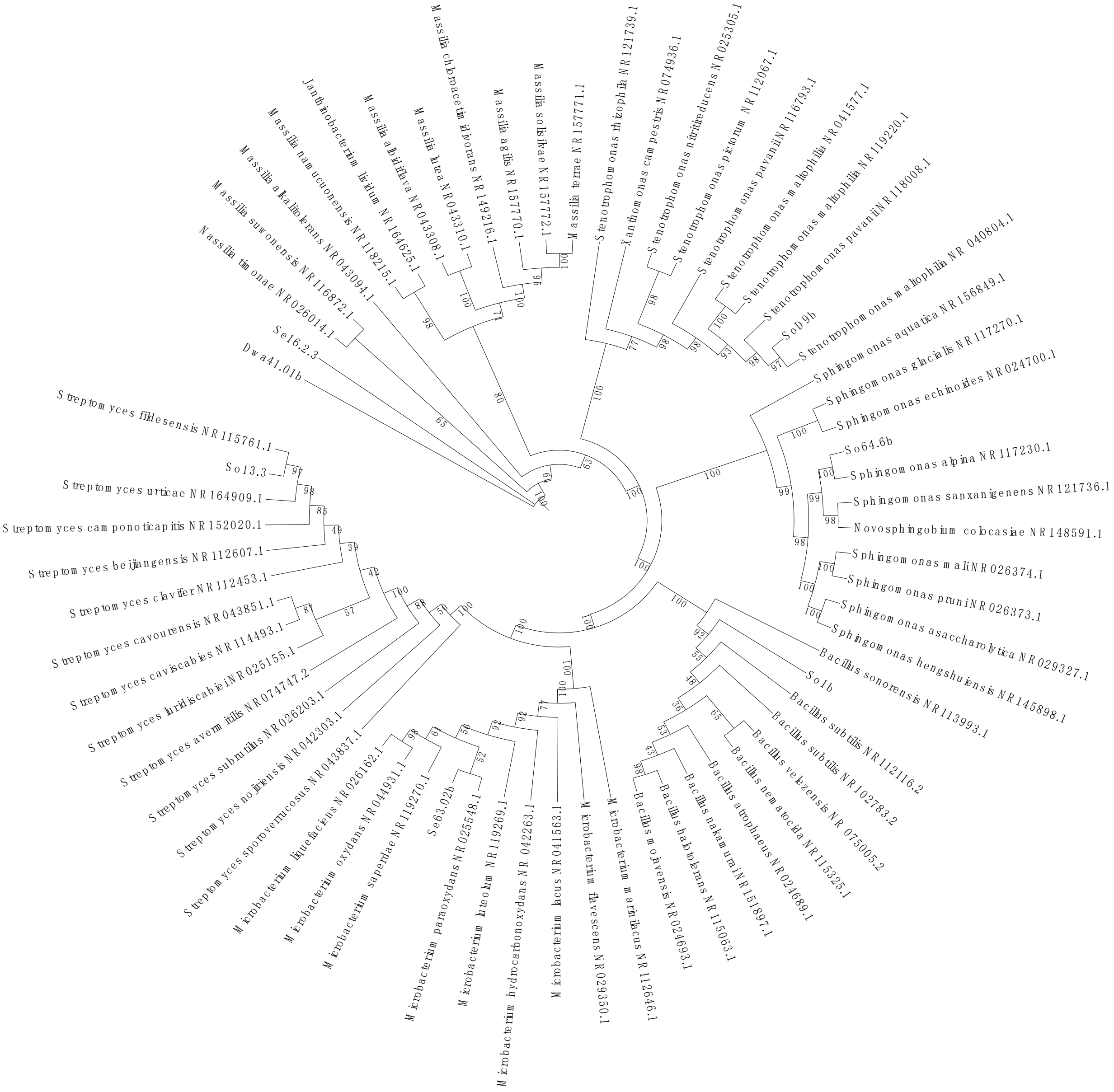

Figure S2. Maximum-likelihood phylogenetic tree based on complete 16S rRNA gene sequences showing the genetic distances between Antarctic bacterial strains (highlighted in color and bold) and closely related species based on 16S rRNA reference sequences from DDBJ/ENA/GenBank database. Numbers at nodes represent the bootstrap support (%). Accession number of 16S rRNA gene sequences are shown after the species name.

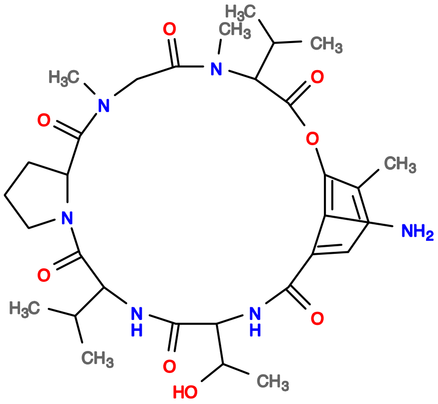

Scaffold 1 predicted from So13.3 gene cluster
$C_{31}H_{46}N_6O_8$
Molecular weight: 630.730

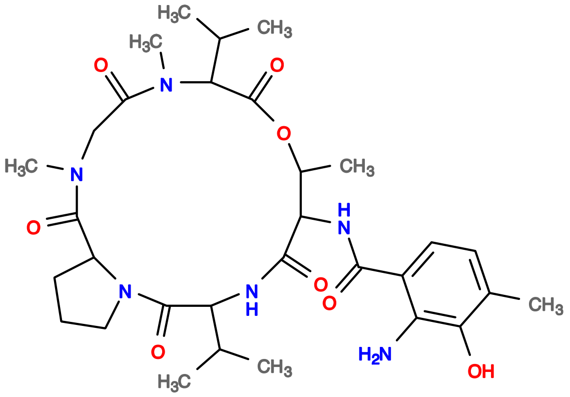

Scaffold 2 predicted from So13.3 gene cluster
$C_{31}H_{46}N_6O_8$
Molecular weight: 630.730

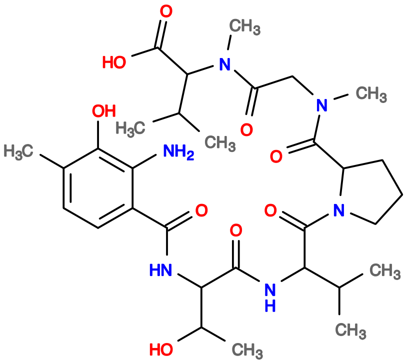

Scaffold 3 predicted from So13.3 gene cluster
$C_{31}H_{48}N_6O_9$
Molecular weight: 648.750

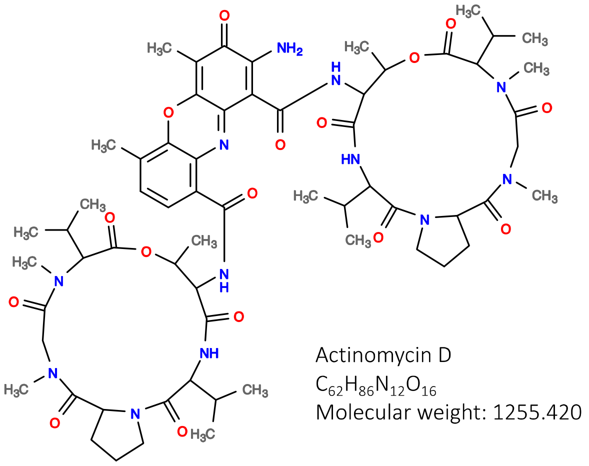

Actinomycin D
$C_{62}H_{86}N_{12}O_{16}$
Molecular weight: 1255.420

Figure S3. Chemical structures predicted for biosynthetic gene cluster similar to Actinomycin D from the Antarctic strain So13.3 genome (Scaffolds 1-3) compared to the structure of the known drug. The predicted structures were based on the core biosynthetic genes using PRISM 4 (http://grid.adapsyn.com/prism/#!/prism).
